# Myocardial fibrosis affects outcome of pulmonary valve insertion after tetralogy repair

**DOI:** 10.1093/icvts/ivad045

**Published:** 2023-03-14

**Authors:** Tom R Karl

**Affiliations:** Queensland Paediatric Cardiac Service and Queensland Pediatric Cardiac Research, University of Queensland, Brisbane, QLD, Australia

**Keywords:** Tetralogy of Fallot, Pulmonary valve, Myocardial fibrosis

The transannular patch repair of tetralogy of Fallot never seems to go out of style, despite the availability of other valve function-preserving techniques. I read with great interest the current article from the highly credible and experienced team at Fuwai Hospital (National Center for Cardiovascular Disease, Beijing, China) [1]. As acknowledged by the reporting team, there is already extensive MRI data supporting the thesis that extensive fibrosis occurs in postoperative tetralogy cases with longstanding pulmonary insufficiency and may be either a cause of effect of right ventricular dilation. The patients in this study met standard criteria for pulmonary valve insertion based on clinical presentation and MRI derived ventricular volumetric measurements. The novelty of this analysis is the attempt to correlate the probability of postoperative adverse events with the actual degree of fibrosis, using directed myocardial biopsies. Despite the difficulties in performing such a study (sampling sites, patient selection, statistical treatment, generalizability, etc.), I think the authors have achieved what they set out to do. Their data do support the conclusions, namely that histological myocardial fibrosis was correlated inversely with biventricular systolic function. A higher degree of myocardial fibrosis and older age at PVR were independent risk factors for adverse cardiac events after PVR in patients with repaired tetralogy of Fallot. The main question is how to use this information, which strongly supports what we already know from MRI data. Clearly, intraoperative information is not useful for patient selection, and it seems unlikely that management strategies could be directly affected either in a way that would prevent the occurrence of adverse events. So, while the article stands on its own scientific merits, the translational importance of the results remains to be seen. Having said that, the study is thoughtful and well-conducted study, and I certainly enjoyed reading and reviewing the article. As with any good study, more questions are raised than answered.
